# Single-Cell Transcriptome Profiling Signatures and Alterations of Microglia Associated With Glioblastoma Associate Microglia Contribution to Tumor Formation

**DOI:** 10.3389/pore.2022.1610067

**Published:** 2022-05-25

**Authors:** Hailong Xia, Lei Deng, Shu Meng, Xipeng Liu, Chao Zheng

**Affiliations:** ^1^ Department of Neurosurgery, Chongqing Red Cross Hospital (Jiangbei District People’s Hospital), Chongqing, China; ^2^ Department of Neurosurgery, Bishan District People’s Hospital, Chongqing, China; ^3^ Internal Medicine, Chongqing Red Cross Hospital (Jiangbei District People’s Hospital), Chongqing, China

**Keywords:** GBM, single-sell RNA-seq, microglia, tumor immune microenvironment, aged microglia

## Abstract

Glioblastoma (GBM), which occasionally occurs in pediatric patients, is the most common tumor of the central nervous system in adults. Clinically, GBM is classified as low-grade to high-grade (from 1 to 4) and is characterized by late discovery, limited effective treatment methods, and poor efficacy. With the development of immunotherapy technology, effective GBM treatment strategies are of great significance. The main immune cells found in the GBM tumor microenvironment are macrophages and microglia (MG). Both these monocytes play important roles in the occurrence and development of GBM. Macrophages are recruited during tumorigenesis, whereas MG is present in the brain during embryonic development. Interestingly, the accumulation of these monocytes is inversely proportional to the survival of adult GBM patients but not the pediatric GBM patients. This study used single-cell RNA-seq data to reveal the heterogeneity of MG in tumor lesions and to explore the role of different MG subtypes in the occurrence and development of GBM. The results may help find new targets for immunotherapy of GBM.

## Introduction

GBM is the most common and infiltrating type of primary brain tumor, with a global incidence of approximately 70,000–100,000 per year. It is characterized by hidden onset, high morbidity, high fatality, high recurrence, and low cure rates [1]. Even with active therapy, GBM is extremely prone to relapse and has poor prognosis. The median survival time of patients is usually between 15–19 months, and the 5-year survival rate is only 5%. Approximately 180,000–600,000 young and middle-aged people die of GBM worldwide each year [2, 3].

In the past decade, researchers have found that the tumor microenvironment has a notable impact on tumorigenesis and tumor development [4]. The interactions and mutual influence between non-tumor and tumor cells have gradually attracted attention and led to more in-depth discussions. Owing to the presence of the blood-brain barrier, the tissue environment in which GBM exists is very different from that of other tumors. Most GBM tumors are characterized by immune tolerance. MGs, macrophages, and T cells constitute the immune microenvironment [5, 6].

Malignant tumor and immune cells in the immune microenvironment are regulated. Inflammatory infiltrating cells, including macrophages and MG, account for more than half of the total immune cells in the GBM immune microenvironment [4]. They are considered important in inducing tumor invasion and growth by secreting CCL5, IL-113, TGF-β, EGF, IL-6, and platelet-derived growth factors [7, 8]. During its immune response to GBM, MG can also release soluble factors to promote tumor migration. It is believed that malignant tumor cells can secrete chemokines (CCL2 and CXCL2) [9, 10] to recruit macrophages and MG, which gather around tumor cells, thereby promoting tumor formation and evading immune cell attack. MG is a source of yolk sac myeloid precursor cells.

In GBM, the mechanism through which MG differentiates into subgroups with different roles is not fully understood. Previous studies have shown that the loss of MG induced in different ways can inhibit the growth of malignant gliomas [11–13]. *Gpnmb* and *Spp1* genes have also been implicated in the accumulation of disease-related MG and MG-related cancer cell proliferation, and they are associated with poor prognosis of human GBM [14]. The tetracycline analog minocycline can block the activation of MG and reduce tumor growth in a GBM mouse model [15, 16]. In a mouse brain tumor slice model, antibodies against the MG Toll-like receptors (TLRs) target the tumor size [17]. Unfortunately, human clinical trials targeting MGs have not yielded satisfactory results. At the same time, no markers have been identified that can distinguish tumor-associated MGs from other MG subgroups. Accordingly, analyzing single-cell sequencing data to explore the heterogeneity of MGs in the immune microenvironment of GBM can help determine the differentiation mechanism of tumor-related MG cells during tumor formation. This will provide a potential target for molecular diagnostics and clinical therapy of GBM.

## Materials and Methods

### Data Collection

Single-cell RNA-seq data from 10 adult and pediatric patients with IDH-wildtype glioblastomas were downloaded from the Gene Expression Omnibus (GEO) database (dataset NO. GSE131928). The old and young mice brain single-cell RNA-seq data were also downloaded from the GEO database (dataset NO. GSE147693).

### Single-Cell RNA-Seq Data Processing

The Cell Ranger software (v3.3.0) provided by 10x Genomics contains raw data with barcodes after single-cell sequencing. The STAR (v0.3.7) tool was used to map reads to the genome and transcriptome and aggregate the data in the samples to generate normalization data for generating a gene expression count matrix corresponding to the cell. We used the Seurat R package to process the unique molecular identifier (UMI) count matrix (v3.0) [18]. To remove low-quality cells and possible multiple captures, we filtered out cells with a limit of +/− 2 times the number of UMI/genes beyond the average value, assuming that the UMI/gene of each cell has a Gaussian standard deviation. After checking the cell distribution ratio for mitochondrial gene expression, Based on the distribution of mitochondrial gene expression, we further discarded >20% of the cells. After applying these quality control standards, we normalized the filtered matrix in Seurat to obtain normalized counts. Principal component analysis (PCA) was performed to reduce matrix dimensions. The cells are clustered based on the graph-based clustering method and visualized in two dimensions using Uniform Manifold Approximation and Projection (UMAP). Marker genes can simultaneously test the average expression and percentage of expressed cells to identify genes that are significantly differentially expressed between the clusters. For cell type identification, the singleR package (v1.4.1) [19] was used to assist in the determination based on the “HumanPrimaryCellAtlasData” parameter.

### Pseudotime Analysis

We used Monocle and Monocle3 packages (http://cole-trapnell-lab.github.io/monocle-release/) to set a pseudo-chronological sequence of cell development [20]. First, we used the importCDS function in Monocle to convert the original count matrix in the Seurat object into the CellDataSet format and determine the differentiation trajectory between cells. Then, the Monocle3 package was used to infer the pseudo-time trajectory and differentiation direction of cell development.

### Differential Expression Analysis

Differential expression analysis was performed using the MetaDE package (v1.30.1) [21]. Significantly differentially expressed genes were identified using the meta-analysis method and Bonferroni correction (adjusted *p*-value).

### Gene Ontology Term and Pathway Enrichment Analysis

We took the significantly different genes obtained from the Single-cell RNA-seq analysis as the gene set and used the clusterProfile package (v3.18.1) [22] to perform GO and KEGG function and pathway enrichment analysis. The column chart displays the KEGG pathway enrichment results and the original chart displays the GO term enrichment analysis. The results show the enrichment results of the top ten enrichment scores and the presence of cross genes in the enriched set in the form of a network.

### PPI-Network Analysis

The differentially expressed genes between the single-cell clusters were placed into the protein interaction database (STRING: functional protein association networks online analysis software), the differential gene interaction (from curated databases and experimentally determined) relationship network was extracted, and Cytoscape (v3.6.1) was used to perform protein interaction network analysis and find the key network nodes by combining GO and KEGG enrichment results.

### TCGA Clinical and Sequencing Data Analysis

Connecting to the TCGA database through cBioPortal (www.cbioportal.org), we downloaded the RNA-seq data of 543 patients and the survival analysis data of 147 patients in the glioblastoma (TCGA, Cell 2013) gene set. We then performed mRNA co-expression analysis and analyzed the gene expression differences corresponding to the survival rate analysis.

## Result

### Single-Cell RNA-Seq Identifies Multiple Cell Population in 10 GBM Samples

**FIGURE 1:**
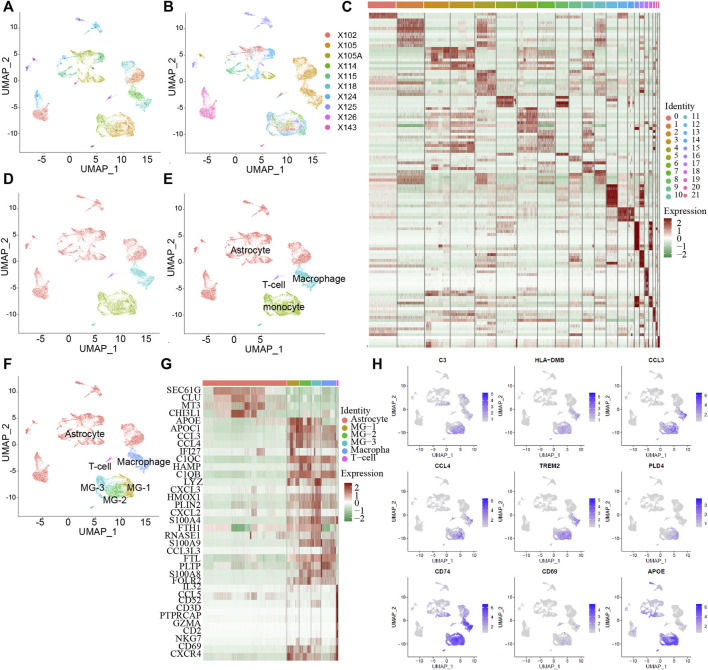
The subtype of MG cells in GBM tumor microenvironment. Integrated single-cell RNA-Seq analysis of human GBM tumor cells. All ten samples were analyzed using canonical correlation analysis with the Seurat R package. Cells were clustered using a graph-based shared nearest neighbor clustering approach and visualized using a UMAP plot. **(A,B)** All cells were clustered in 21 clusters from 10 patients. **(C)** 21 cluster’s marker genes expression heatmap. **(D)** All cells were characterized into 4 cell types. **(E)** Automatically identify cell types with the singleR package. **(F)** Combined with the GBM tumor microenvironment, identify the cell type and the MG cell subtype. **(G)** Heatmap of marker genes expressed in different cell types. **(H)** Some of MG cell associate genes are report by the previous studies and the current MG especially expressed genes.

To study the influence of MG in the tumor microenvironment of human GBM on tumorigenesis and development, we downloaded 10X single-cell RNA-seq data from 10 GBM samples (Figure 1A) from the GEO database. Among them, nine samples were from adults and one sample was from a minor. After data quality control and principal component analysis (PCA) (Supplementary Figure S1), all cells were classified into 21 clusters (Figure 1A), combined with the marker genes of cells in the HumanPrimaryCellAtlasData database, and they were divided into five cell types (Figure 1D). Malignant tumor cells accounted for the largest percentage of cells, followed by macrophages, monocytes, T cells, and endothelial cells. Based on previous studies on the microenvironment of GBM tumors and the marker gene of each cluster (Figure 1C), the monocyte cluster was further divided into three subsets of MGs (Figure 1F). Analysis of the marker genes (Figure 1G) of the final six cell types revealed that the three subsets of MG-1, MG-2, and MG-3 have the same markers (Figure 1H), including *CCL3*, *CCL4*, and *CXCL2*, which have been reported to be significantly elevated in MG.

### GBM Associated MG Cells Transcriptomic Alteration Analysis

**FIGURE 2:**
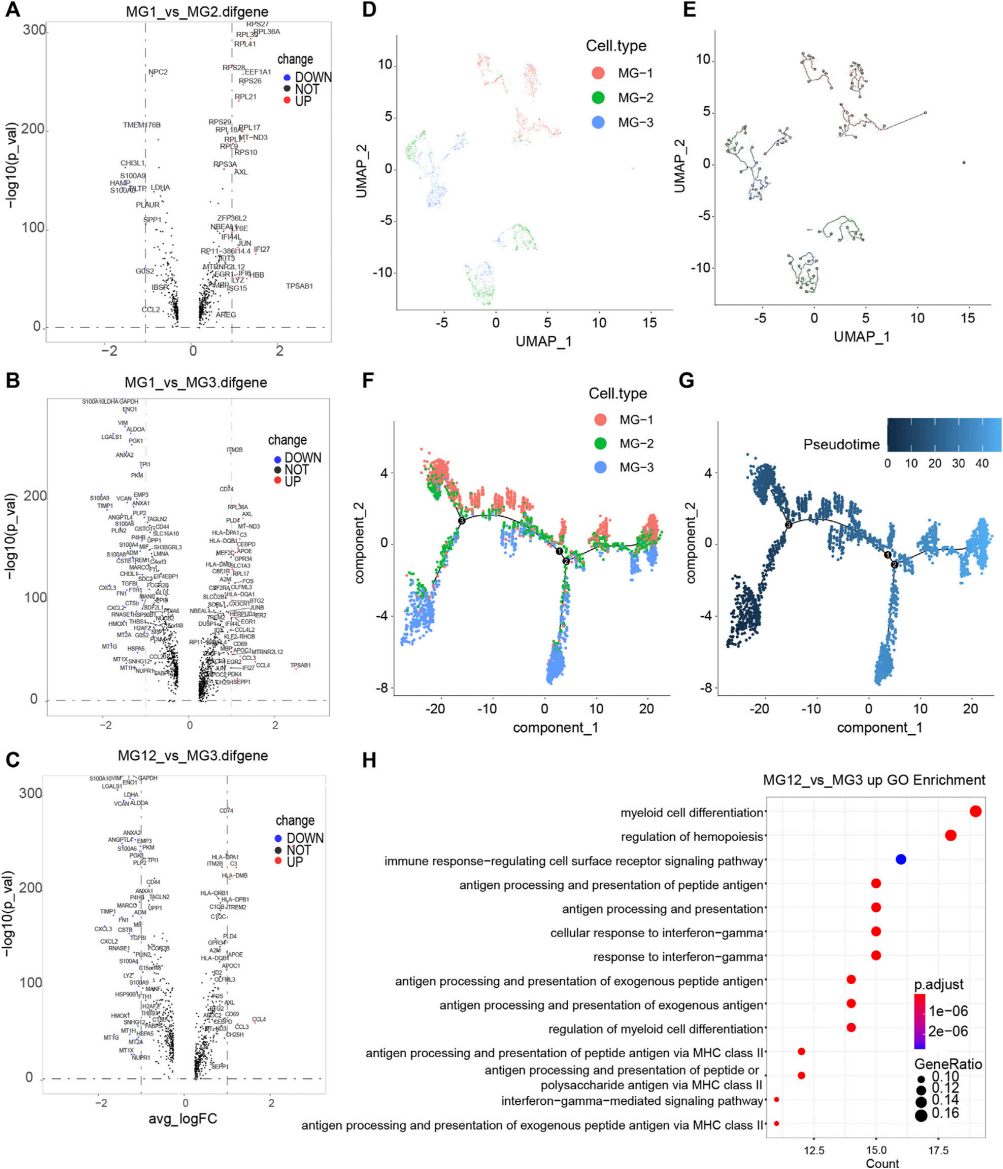
Genetic analysis of differences among subtypes of MG cells in GBM tumor microenvironment. **(A–C)** Volcano plot of different genes between every two subtypes of MG cells. Significantly different genes (logFC > 1 and −log10 (p_val) > 20) are red dots (with gene names), while black dots represent those that are not significant; the x-axis represents the log2 (fold change) and the y axis is the −log10 (p_val). **(D,E)** Monocle relies on machine learning technology, Reversed Graph Embedding, to construct single-cell trajectories. **(F,G)** Trajectory inference by monocle3, the Pseudotime value of a cell, is the distance from its position along the branch to the root (relatively). **(H)** Functional enrichment analysis was performed using GO enrichment with the significantly upregulated genes in MG-12 compare to MG-3. The top 20 significantly enriched GO processes are shown.

**FIGURE 3:**
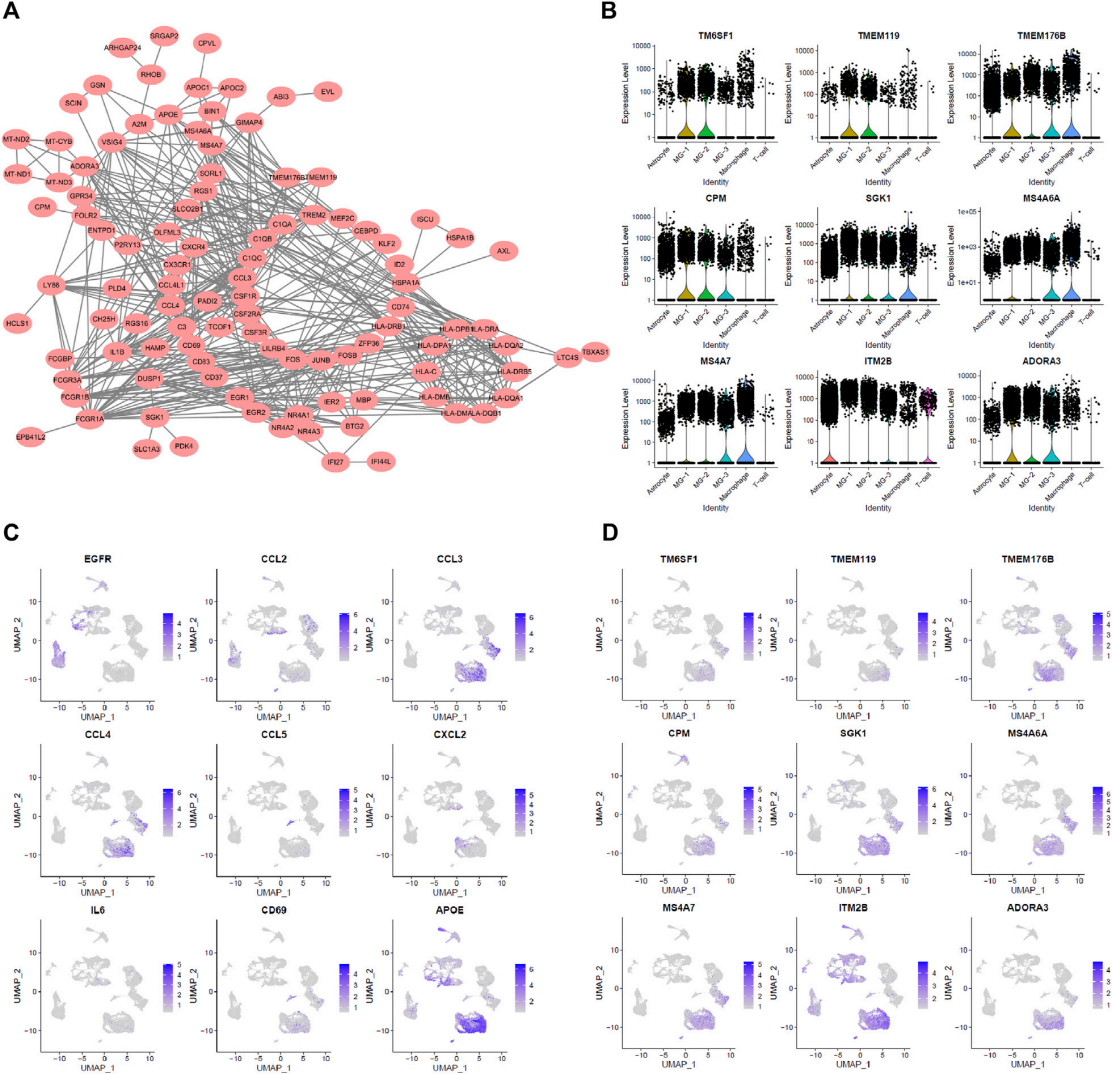
Characteristic of MG-12 up-regulation genes. **(A)** PPI-Network representing related MG-12 up-regulation genes. **(B)** Cell chemotaxis-related genes. **(C,D)** Myeloid cell differentiation GO term genes expression in all cell types.

First, the three subsets of MG cells were analyzed for differential expression in pairs (Figures 2A–C; Supplementary Figure S2). The results showed that the overall difference between MG-1 and MG-3 was large, whereas the difference between MG-1 and MG-2 was small. In the analysis of intercellular development trajectory (Figures 2D,E), the distance between MG-1 and MG-3 was greater than that between MG-1 and MG-2. The pseudo-time trajectories (Figures 2F,G; Supplementary Figures S3A,B) showed that MG-2 may be the intermediate stage of MG-1 and MG-3. Finally, we performed GO and KEGG function and pathway enrichment analyses (Figure 2H; Supplementary Figures S3C,D) of the genes showing significant differential expression between the MG-1 and MG-2 combination and MG-3. The results showed that the most significant GO function terms were the myeloid cell chemotaxis-related genes (Figures 2B, 3A) and many inflammatory and immune response pathways (Supplementary Figures S3C,D). In particular, the upregulated genes in MG-1 and MG-2, compared to those in MG-3 (Figure 3A), were significantly enriched in multiple genes related to myeloid cell chemotaxis, and the expression of these genes in MG cells was significantly higher than that in other cell types (Figures 3B–D). Integrating the results of MG cell heterogeneity and pseudo-time analysis, we hypothesize that MG in the GBM tumor microenvironment produces inflammatory pathways and activates immune response pathways (especially MHC-II). During the analysis, we found several significantly altered genes (*ZFP36*, *NFKBID*, *JUNB*, *FOS*, and *FOSB*) (Figure 3B), which are not only related to the chemotaxis of myeloid cells but are also closely related to cell senescence.

### MG Cells Alteration Between Aged and Young Mouse Brain Single-Cell RNA-Seq

**FIGURE 4:**
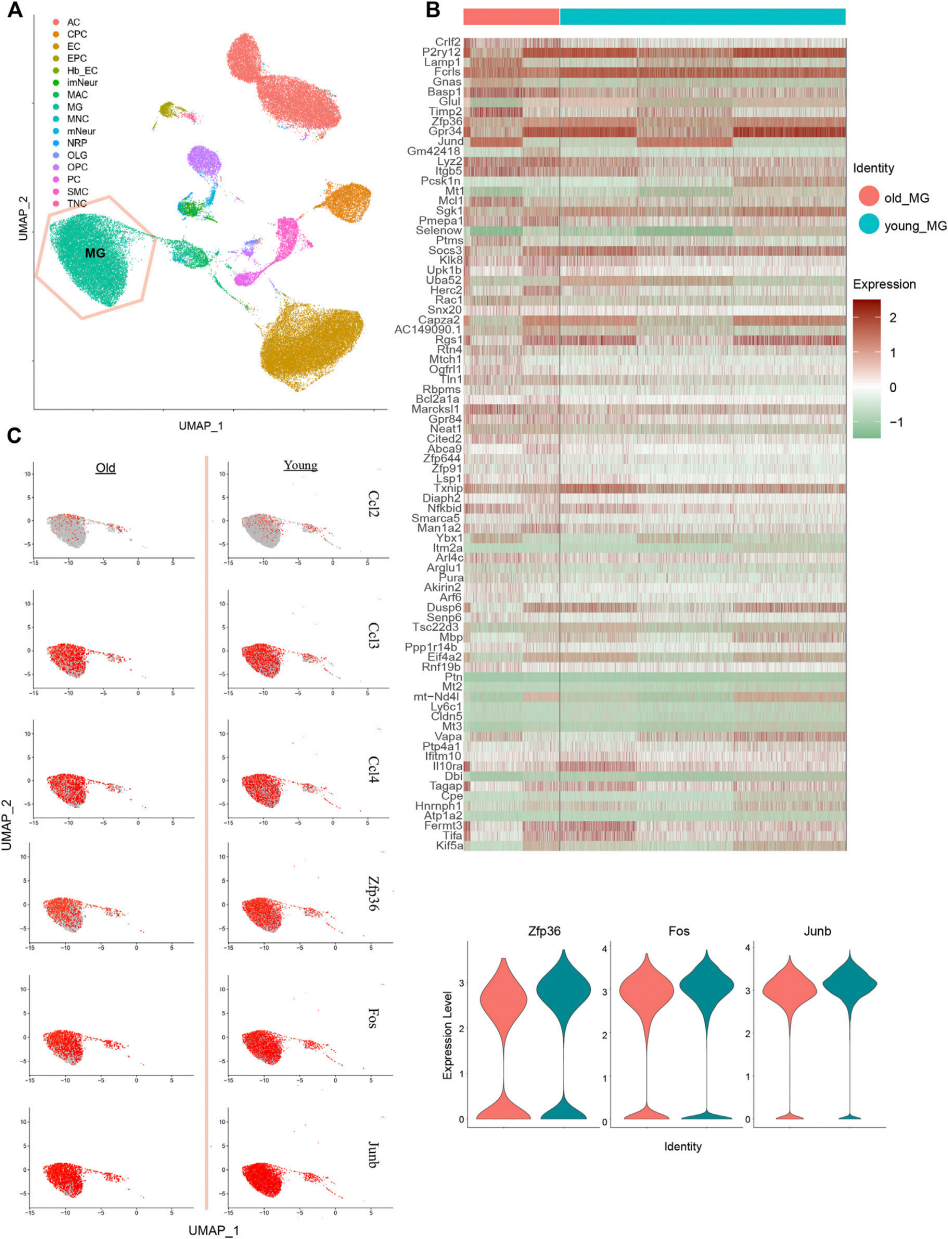
Performance of MG-12 up-regulation genes in MG cells from old and young mice brain single-cell RNA-seq data. **(A)** Identification of cell types on UMAP plot. **(B)** Heatmap of the top 100 different genes between old and young MG cells. **(C)** UMAP plot and violin plot show the relative expressions of old and young MG cells in MG-12 up-regulation genes.

Based on the key differential genes and pathways we identified, we found that MG only has a cumulative effect in adult GBM; this is consistent with the study by Engler et al. We Further focused on what happens in MG cells during cell senescence. To compare the changes in MG cells during the aging process and those during the formation of GBM, we downloaded single-cell RNA-seq data of young and old mice whole brains from the GEO database. The MG cells were extracted based on cell typing, as in the original paper (Figure 4A). Further analysis of the differential genes in the expression profiles between old and young MG cells (Figures 4B,C), and the differential genes of GBM-related MG subsets, such as Zfp36 and Nfkbid, were found in the age differences of top 200 genes (Figure 4B). Changes in related genes were further verified in terms of the expression level and proportion of expressing cells (Figure 4C). ZFP36, as an inhibitor of the cellular senescence pathway, and NF-κB, as an activator, can directly regulate the expression of SASP-related 6 genes, thereby regulating cellular senescence. *ZFP36* and *NFKBID* decreased when MG cells aged, which is probably the key factor that causes only adult GBM to be affected by the number of MG cells.

### Specific Manifestations of Genes Associated With MG Cell Heterogeneity in Clinical Data

**FIGURE 5:**
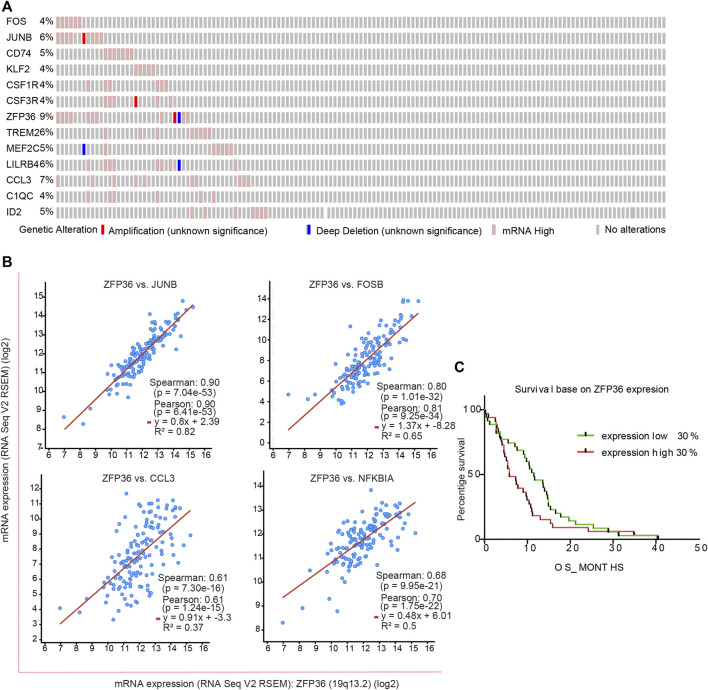
Characteristic of MG-12 up-regulation genes in clinical data from TCGA database. **(A)** Comprehensive status of important genes in clinical samples. **(B)** The significance fitting curve of co-expression between genes. The red curve **(C)** The survival curve was significantly correlated with the expression of the *ZFP36* gene (*p* = 0.0398, Gehan-Breslow-Wilcoxon test).

To understand the important role of *ZFP36* and other key genes in the survival and prognosis of GBM, we analyzed the bulk RNA-seq data of tumor samples and their corresponding survival data by downloading the “Glioblastoma (TCGA, Cell 2013)” data. Among the 543 tumor samples, a considerable portion showed high mRNA levels of these genes (Figure 5A). Almost all the above genes were co-expressed with a significant positive correlation (Figure 5B; Supplementary Figure S4). Further analysis of the correlation between ZFP expression levels and GBM survival (Figure 5C) revealed significant difference (*p* < 0.05) between high and low ZFP expression levels. Analysis of clinical data further verified that *ZFP36* and other MG heterogeneity-related genes play an important role in the survival and prognosis of GBM.

## Discussion

The research for GBM therapy has been progressing slowly. Drugs against GBM are limited by the blood-brain barrier. Various studies have shown that the antitumor immune response of GBM is modulated. This indicates the feasibility of immunotherapy for GBM treatment. Like many other malignant tumors, GBM highly expresses typical immunosuppressive factors, such as *PD-L1*; A lot of progress has been made in immunotherapy targeting T cells [23–25]. However, owing to the blocking effect of the blood-brain barrier, the immune cell composition in the tumor microenvironment of GBM is significantly different from that of other tumors. This also suggests that it may be more meaningful to target GBM tumor-related macrophages and MG for immunotherapy of GBM.

The development of single-cell sequencing technology provides better methods and possibilities for studying cell heterogeneity and cell state transformation. In this study, we discovered a GM state identification method that is different from the traditional M1/M2 state model. In our cell state identification, we did not observe a difference in the expression of the typical features of M1/M2 (iNOS and Arginase1) [26]. Using the pseudo-time analysis method based on different algorithms, we found that MG-1 and MG-3 from our three classifications should be the two most distant populations. However, owing to the limitation of the depth of single-cell sequencing, we did not observe numerous differences between the two cell populations in the single-cell data. Fortunately, we are still exposed to a series of important differential genes, such as *ZNF36*, in gene set enrichment analysis. *ZNF36* is associated with the MAPK and TNF pathways and possesses complex negative feedback regulation mechanisms [27]. These two important pathways have been reported in many studies to be closely related to GBM [28–30], especially with respect to inflammation (MHC II) [31] and immune activation [25, 32]. In addition, we found that some genes such as *ZNF36*, *AP-1*, and *CCL3* are closely related to cell differentiation [33–36], especially the differentiation of myeloid monocytes as chemokines. This result suggests that chemotaxis between different types of MG cells is likely to be affected by the pathways in which genes such as *ZNF36* are located, and that this mechanism is affected by stress from the tumor environment, such as inflammatory factors.

Given the simultaneous appearance of key genes, such as *ZNF36*, *NFKBID*, and *IL-6*, we focused on the cellular senescence pathway [37]. There are large differences between adult and pediatric GBM, resulting in many therapy programs for adult GBM that cannot be used for children. The enrichment of these genes related to the cellular senescence pathway may remind us that the difference between adult and pediatric patients is important for GBM tumor microenvironment. We identified MG cells in old and young mice through whole-brain single-cell sequencing data. Changes in MG cells during the aging process were analyzed. Perhaps because of the technical limitations of the depth of single-cell sequencing or the experimental protocol, we did not enrich the pathway in the aged and younger groups. It is worth noting that the main differential genes found in the tumor-related MG cell state differential gene set were still found to appear in the elderly related differential gene set. This result suggests that changes in MG cells during the aging process may have a huge impact on the tumor microenvironment of GBM.

To verify the criticality of the genes found in GBM, we performed co-expression analysis and survival analysis in the RNA-seq data of human GBM tumor samples. Except for these results, these genes not only showed very good consistent expression in tumor samples, the expression level of *ZFP36* was also significantly negatively correlated with patient survival.

## Data Availability

Publicly available datasets were analyzed in this study. This data can be found here: dataset No. GSE131928, dataset No. GSE147693 Glioblastoma (TCGA, Cell 2013).

## Supplementary Material

Supplementary Figure S1 QC and PCA analysis of single-cell RNA-seq data. **(A)** Overview of all cell types of variable genes in the 10 samples highlighting the top-10 significant variable genes. **(B)** The top-20 principal components and their significance. **(C)** gene set that has greater influence in the top-9 principal components. **(D)** Heat-map based on a single principal component.

Supplementary Figure S2 Marker genes of MG cell subtypes in GBM tumor microenvironment. **(A–C)** MG-1, MG-2, and MG-3 cluster marker genes are compared to each other.

Supplementary Figure S3 Characteristic analysis of the subtypes of MG cells. **(A)** Genes with dispersion (variation) coefficients higher than the set value were selected for developmental trajectory analysis. **(B)** The stage of cells in Figures 2F,G. **(C,E)** Downregulated gene set KEGG pathway enrichment analysis result, top-10 pathway and their relationship (lines means that there are overlap genes between the two pathway). **(D,F)** Upregulated gene set KEGG pathway enrichment analysis result, top-10 pathway and their relationship (lines means that there are overlap genes between the two pathway).

Supplementary Figure S4 ZFP36 signature co-expressed genes in TCGA GBM RNA-seq. **(A–F)** *CD83*, *CCL4*, *CCL4L1*, and *NFKB1* are co-expressed with *ZFP36* in clinical RNA-seq.

## Abbreviations

CNS, the central nervous system; GBM, glioblastoma; HLA, human leukocyte antigen; MG, microglia; MHC-II, major histocompatibility complex classII; MMP, matrix metalloprotease; OS, overall survival; PCA, principal component analysis; TGF-β, transforming growth factor β; UMAP, uniform manifold approximation and projection.
