# Supplementary figures and images for: Single-Cell Transcriptome Profiling Signatures and Alterations of Microglia Associated With Glioblastoma Associate Microglia Contribution to Tumor Formation
Source: Pathol Oncol Res. 2022 May 25;28:1610067. doi: 10.3389/pore.2022.1610067 (PMC9176381; doi:10.3389/pore.2022.1610067)

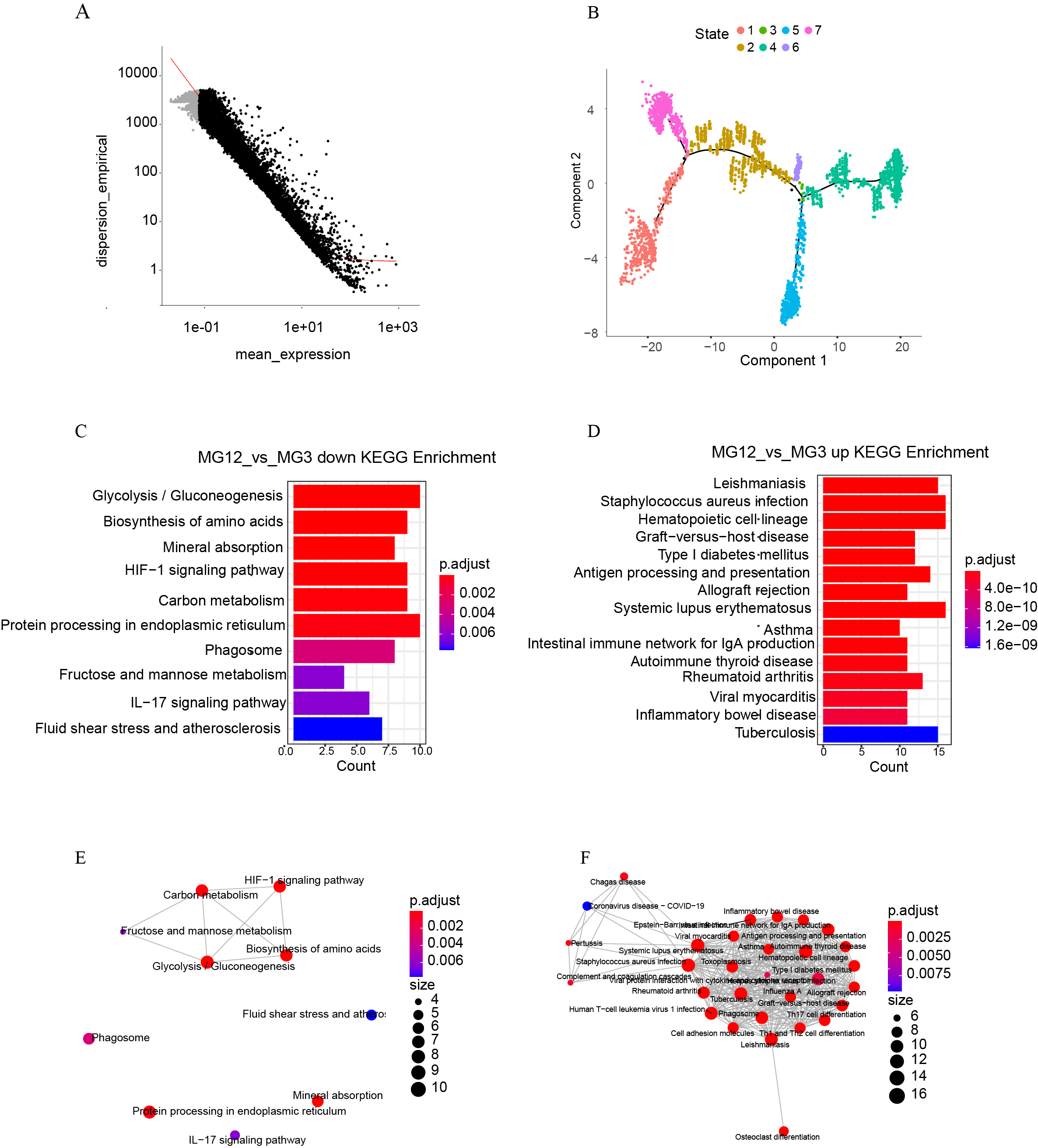

Supplement: Supplementary file 1 [file Image3.TIF]

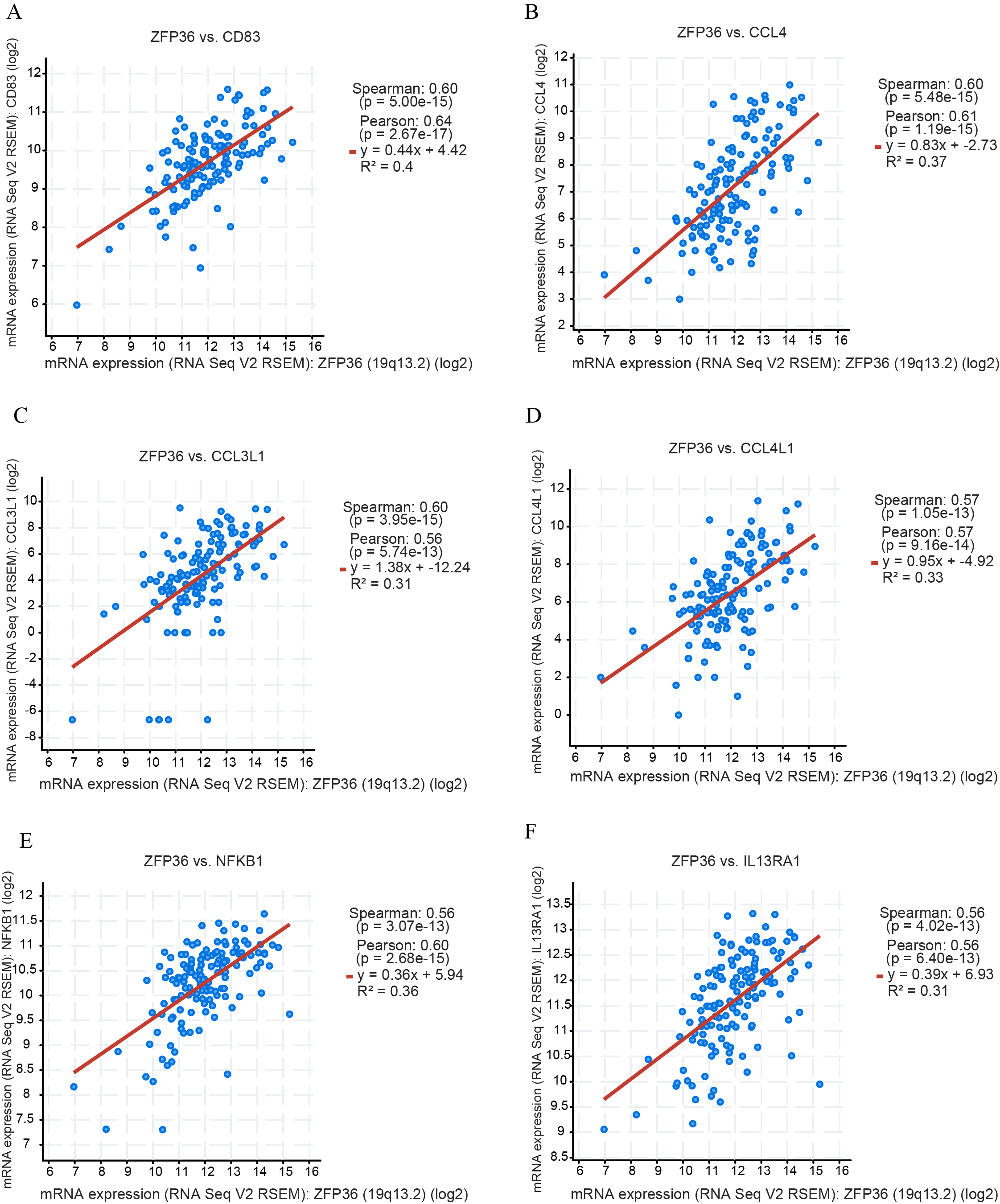

Supplement: Supplementary file 2 [file Image4.TIF]

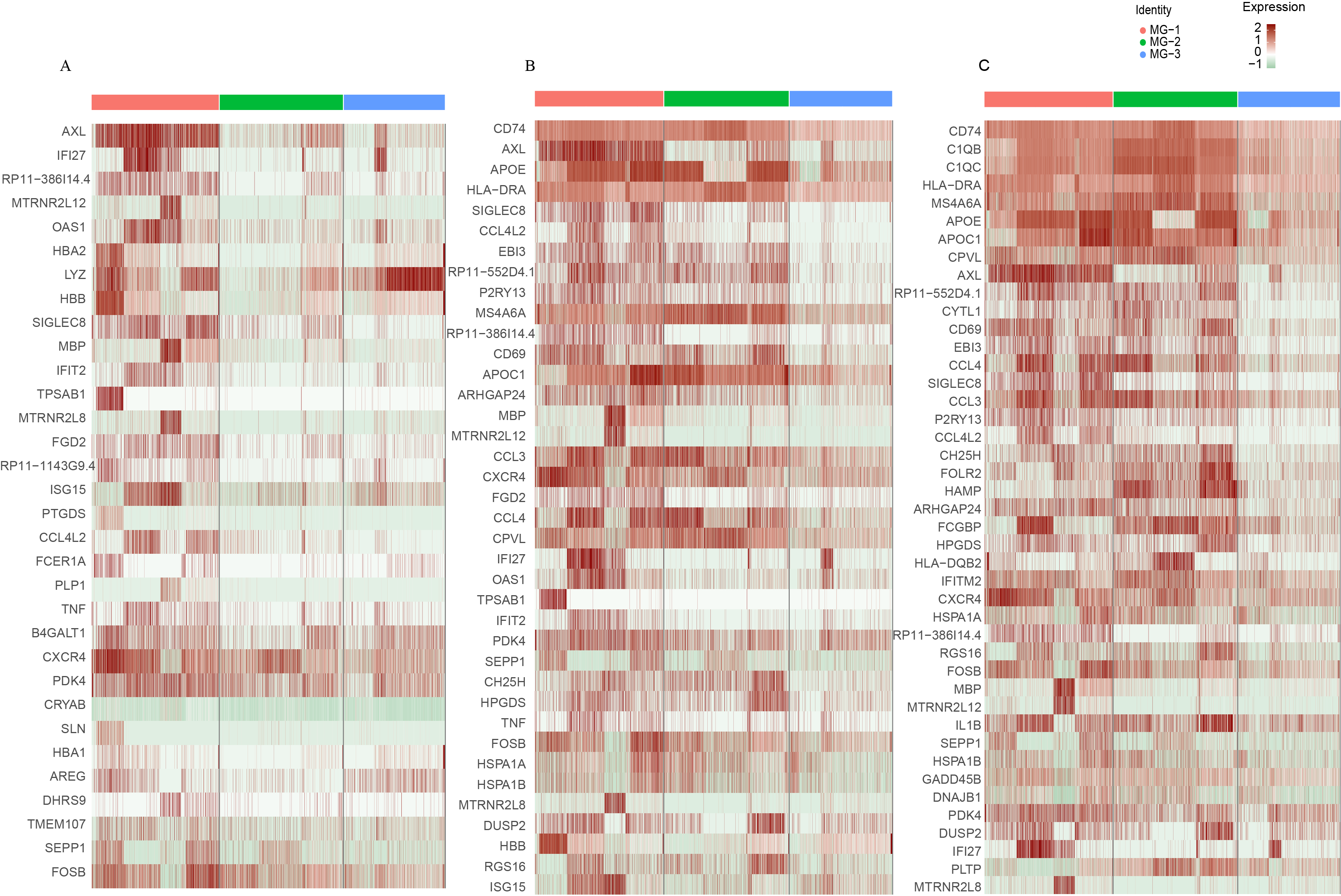

Supplement: Supplementary file 3 [file Image2.TIF]

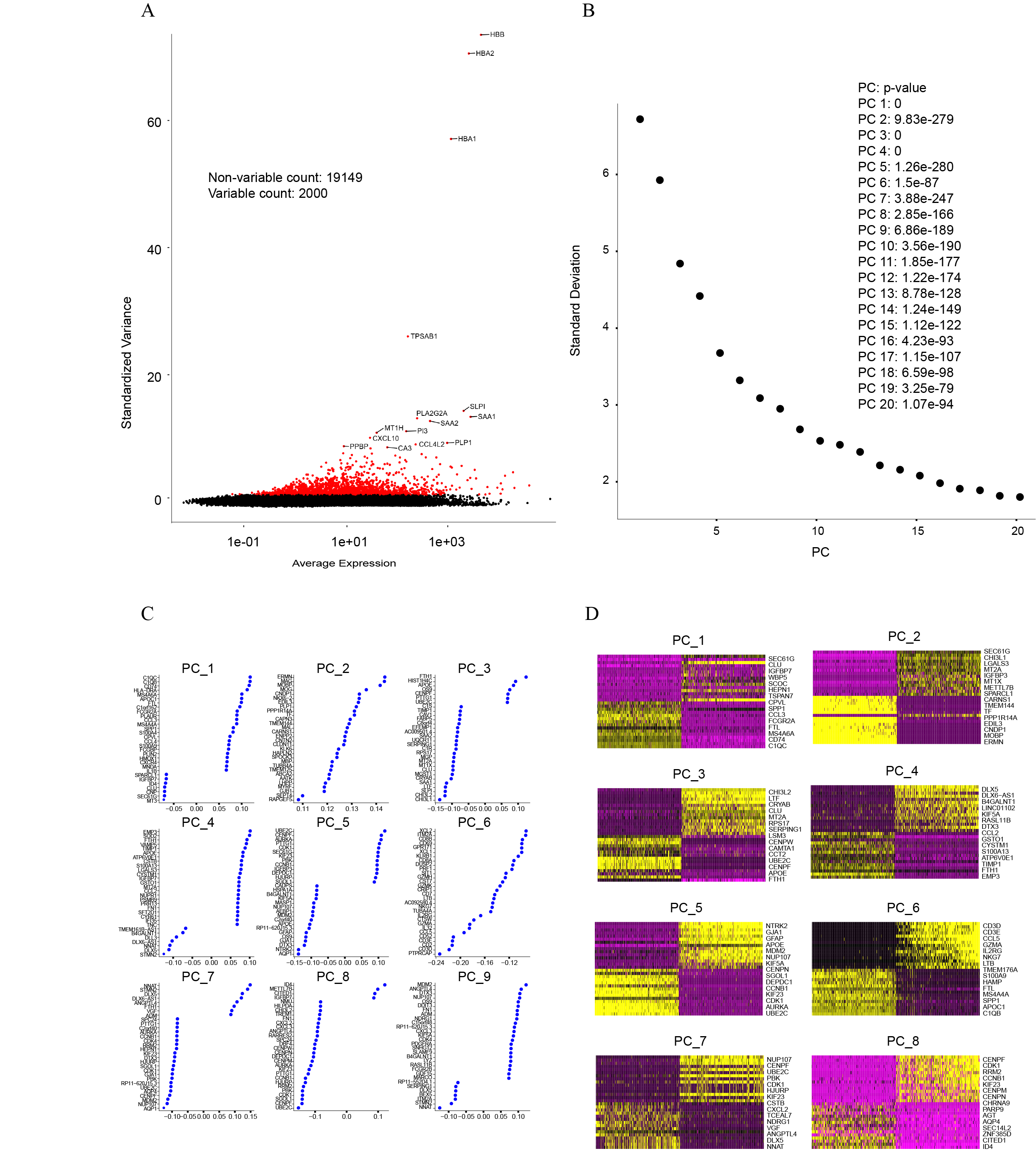

Supplement: Supplementary file 4 [file Image1.TIF]
